# Patients with chronic fatigue syndrome performed worse than controls in a controlled repeated exercise study despite a normal oxidative phosphorylation capacity

**DOI:** 10.1186/1479-5876-8-93

**Published:** 2010-10-11

**Authors:** Ruud CW Vermeulen, Ruud M Kurk, Frans C Visser, Wim Sluiter, Hans R Scholte

**Affiliations:** 1 CFS/ME and Pain Research Center Amsterdam, Waalstraat 25-31, 1078 BR Amsterdam, The Netherlands; 2 Department of Neurology, Erasmus MC University Medical Center, Rotterdam, The Netherlands; 3 Department of Neuroscience, Erasmus MC University Medical Center, Rotterdam, The Netherlands

## Abstract

**Background:**

The aim of this study was to investigate the possibility that a decreased mitochondrial ATP synthesis causes muscular and mental fatigue and plays a role in the pathophysiology of the chronic fatigue syndrome (CFS/ME).

**Methods:**

Female patients (n = 15) and controls (n = 15) performed a cardiopulmonary exercise test (CPET) by cycling at a continuously increased work rate till maximal exertion. The CPET was repeated 24 h later. Before the tests, blood was taken for the isolation of peripheral blood mononuclear cells (PBMC), which were processed in a special way to preserve their oxidative phosphorylation, which was tested later in the presence of ADP and phosphate in permeabilized cells with glutamate, malate and malonate plus or minus the complex I inhibitor rotenone, and succinate with rotenone plus or minus the complex II inhibitor malonate in order to measure the ATP production via Complex I and II, respectively. Plasma CK was determined as a surrogate measure of a decreased oxidative phosphorylation in muscle, since the previous finding that in a group of patients with external ophthalmoplegia the oxygen consumption by isolated muscle mitochondria correlated negatively with plasma creatine kinase, 24 h after exercise.

**Results:**

At both exercise tests the patients reached the anaerobic threshold and the maximal exercise at a much lower oxygen consumption than the controls and this worsened in the second test. This implies an increase of lactate, the product of anaerobic glycolysis, and a decrease of the mitochondrial ATP production in the patients. In the past this was also found in patients with defects in the mitochondrial oxidative phosphorylation. However the oxidative phosphorylation in PBMC was similar in CFS/ME patients and controls. The plasma creatine kinase levels before and 24 h after exercise were low in patients and controls, suggesting normality of the muscular mitochondrial oxidative phosphorylation.

**Conclusion:**

The decrease in mitochondrial ATP synthesis in the CFS/ME patients is not caused by a defect in the enzyme complexes catalyzing oxidative phosphorylation, but in another factor.

**Trial registration:**

Clinical trials registration number: NL16031.040.07

## Background

Chronic fatigue syndrome/myalgic encephalopathy (CFS/ME) as a syndrome was defined in consensus meetings by Fukuda et al [1]. Fatigue was the major criterion in the definition. It was described as suddenly occurring, not explained, not caused by exercise, insufficiently relieved by rest and causing a major reduction in physical capacity. The additional symptoms of the syndrome were headache, pain in muscles and joints, unrefreshing sleep, postexertional malaise, sore throat, painful lymph glands and insufficient concentration. The combination of fatigue and four or more of the additional symptoms lasting at least for 6 months, sufficed for the diagnosis of CFS/ME. The main exclusion criterion for CFS/ME, was the presence of a disease that is generally accepted as an actual cause of fatigue.

Several groups of investigators assume that a defective oxidative phosphorylation and subsequent free radical production and oxidative stress play an important role in the pathophysiology of CFS/ME [2-11]. A well accepted way to test ATP synthesis under increased work rate is the cardiopulmonary exercise test (CPET) [12-16]. The ATP synthesis is measured indirectly by testing for oxygen uptake (V'O_2_) as a measure for oxygen consumption (Q'O_2_) in an exercise protocol. Q'O_2 _can be restricted when mitochondria are insufficiently active, or by a restricted supply line of oxygen that consists of the lungs, both ventilation and perfusion, the heart pump, the blood vessels and the hemoglobin concentration in the blood. Modern equipment and algorisms suggested the exclusion of these latter causes of an inadequate Q'O_2_, and supported the likelihood of inactivity of the mitochondrial oxidative phosphorylation. This was also suggested by the finding of a decreased anaerobic threshold in the CFS/ME patients, which is determined by CPET. The anaerobic threshold is the rate of oxygen consumption, when the work rate is reached at which blood lactic acid starts to accumulate, and is due to ATP synthesis from anaerobic glycolysis in muscles. In patients with defects in the oxidative phosphorylation, the anaerobic threshold is also reached at a lower oxygen consumption than in controls [17,18].

In this study we compared the CPET [19] with a direct assay of the oxidative phosphorylation in peripheral mononuclear cells (PBMC), attempting to prove an abnormality of this process in the patients. After 24 h these tests were repeated.

## Methods

Patients who visited the CFS/ME Clinic Amsterdam and healthy sedentary controls were invited for the study. All patients fulfilled the criteria of Fukuda et al [1] for CFS/ME and reported the start of symptoms after an infectious disease. Exclusion criteria were according to Fukuda et al [1]. Contra indications for the CPET were mainly cardiac diseases, hypertension, or the inability to perform the exercise as in arthrosis of the knee. Medication was discontinued 2 weeks before the first test. All subjects performed a CPET on a cycle ergometer (Excalibur, Lode, Groningen, The Netherlands) according to our protocol: 3 min without activity, 3 min of unloaded pedaling, followed by pedaling against increasing resistance until exhaustion (RAMP protocol) and ended by 3 min pedaling with low resistance. The rate of work rate increase was estimated from history, physical examination, gender, weight and height. The participants performed symptom limited exercise tests as described by Wassermann et al. [13]. Verbal encouragement to perform maximally was used during the last phase of incremental exercise. Exhaustion of the leg muscles was the limiting symptom in all participants. The V'E, V'O_2_, V'CO2 and oxygen saturation were continuously measured (Metasoft). The ECG was continuously recorded and blood pressure was measured every 2 min. The CPET was repeated after 24 h. The Respiratory Exchange Rate (RER) was used for validation of the repeated CPET.

The exercise ECG of the subjects was analyzed (by FCV). The anaerobic threshold was determined by the V-slope method.

The participants completed questionnaires among others (not shown) about additional symptoms of CFS/ME (Centers for Disease Control and Prevention Symptoms Inventory - Dutch Language Version (CDC Symptom Inventory-DLV)) [20]. The criterion for fatigue was that at least 4 CFS/ME symptoms must be ≥ 7.5 [20].

All subjects were seen and an ECG was approved by the internist (RMK).

The results of the tests were not available to the participants or the investigators until after the last test was performed by the participant.

Before entry into the study, the nature of the study was explained to the participants and written consent was obtained. The STEG independent ethics committee approved the study. The trial was conducted in accordance with the Declaration of Helsinki (1996 revision) and under the principals of good clinical practice, as laid out in the International Conference on Harmonization document *Good Clinical Practice Consolidated Guideline*.

### ATP synthesis assay of PBMC

PBMC were isolated from 20 mL of blood obtained before each CEPT and anti-coagulated with 0.18% EDTA as described in detail elsewhere [21,22]. For cryostorage in liquid nitrogen, PBMC were suspended at 1 × 10^7 ^cells/mL phosphate-buffered saline, pH 7.4, containing 2 mM EDTA, 10% newborn calf serum and 10% dimethyl sulfoxide. To study mitochondrial function, PBMC were thawed and ATP production via reduction of complex I or II was determined exactly as described [22] except that the cell concentration was decreased to only 5 × 10^4 ^cells per mL incubation medium. A small sample was used to determine citrate synthase (CS) activity according to Srere [23] and protein concentration by the Bio-Rad DC protein assay (Bio-Rad Laboratories) with bovine serum albumine as a standard. The ATP synthesis rate was expressed as nmol ATP synthesized per 30 min per U citrate synthase (CS) or per mg protein.

Plasma creatine kinase (CK) is usually considered a marker of non-specific muscle damage. In the plasma the activity of CK was measured, as a surrogate measure of a lowered oxidative phosphorylation in skeletal muscle. The rationale of this came from early work by Driessen-Kletter et al. [24]. In a group of seven patients with chronic external ophthalmoplegia a high negative correlation of (R = -0.988; *P *= 0.0002) was found between plasma CK 24 h after exercise, and the activity of oxidative phosphorylation via reduction of complex I. Plasma CK was tested by the Clinical Chemistry Laboratory (AKC) of Erasmus MC.

### Statistical analysis

Statistical analyses were conducted using the Statistical Package for the Social Sciences (17.0 for Windows, Chicago, Ill, US). Kolmogorov-Smirnov tests for normality showed that the data were normally distributed. The results were expressed as the mean ± standard deviation (SD). Differences between groups were tested with multivariate or repeated measures Analysis of Variance (ANOVA) where appropriate; correlations were tested with Pearson's correlation test.

## Results

### Patients

Inclusion of patients for the study started in May 2007 and the last CPET was in December 2007. Analysis of the blood samples ended in March 2009. At screening 8 of the 23 patients fulfilled exclusion criteria for the study. In the remaining patients, the results of the male participants were significantly different from females. The low number of male patients prevented separate statistical analysis, therefore only the data of the 15 female participants were reported in this study, together with 15 female healthy controls. Demographic data are presented in Table 1 including the scores for the CDC Symptom Inventory-DLV.

**Table 1:** Age, body mass index and CDC Symptom score in female patients and controls^&^

	Patients n = 15	Controls n = 15
Age (y)	35.5 ± 11.9	35.6 ± 14.0

Body mass index (kg/m^2^)	23.1 ± 5.4	22.7 ± 4.3

CDC Symptom score	59.5 ± 13.1	5.0 ± 4.5*

^&^Data are presented as mean ± SD

*.Statistically significant difference between patients and controls at *P *< 0.01

### CPET1 and CPET2

The V'O_2 _max in CPET1 and CPET2 in the control group were closely related (R = 0.994; *P *< 0.001).

Table 2 summarizes the results of CPET1 and CPET2. At rest the Forced Vital Capacity, the Forced Expiratory Volume (in the first second), the heart rate, oxygen consumption and CO_2 _production were not different between the patient and control group. At the anaerobic threshold the two groups differed for the work rate. (58.6 ± 24.2 W in patients, versus 82.9 ± 29.1 W; *P *= 0.019, 95% CI: -44.3; -4.3), oxygen uptake (12.8 ± 3.0 mL/kg versus 16.7 ± 4.0 mL/kg; *P *= 0.006, 95% CI: -6.52; -1.22) and the ventilatory equivalent for CO_2 _(29.3 ± 2.3 versus 26.9 ± 1.5 in controls; *P *= 0.002, 95% CI: 0.94; 3.86). At maximal work rate, similar differences were seen: work rate (132 ± 30 W versus 188 ± 46 W; *P *= 0.001, 95% CI: -85.5; -27.7), oxygen pulse (9.19 ± 2.18 mL/beat versus 12.43 ± 5.25; *P *= 0.036, 95% CI: -6.25; -0.23) and oxygen uptake (22.3 ± 5.7 mL/kg versus 31.2 ± 7.0; *P *= 0.001, 95% CI: -13.71; -4.16). The results of the second test showed the same differences between the patients and controls (Table 2). The work rate, oxygen pulse and oxygen uptake at the anaerobic threshold and at maximal work rate in the first and second test were closely correlated (paired *t-*test, *P *< 0.001). The oxygen pulse at rest in the first test correlated with oxygen uptake at maximal work rate in the first test (R = 0.63; *P *< 0.001) and in the second test (R = 0.63; *P *< 0.001). The results of the CPET1 and CPET2 showed significant correlations of all measures in the 2 tests (Pearson's test, *P *< 0.001).

**Table 2:** CPET1 and CPET2 in CSF/ME patients versus controls^&^

	CPET1		CPET2	
	Patients n = 15	Controls n = 15	Patients n = 15	Controls n = 15

FVC (L)	3.70 ± 0.73	3.71 ± 0.77	3.71 ± 0.73	3.76 ± 0.72

FEV 1 (L)	2.95 ± 0.61	2.98 ± 0.55	2.94 ± 0.46	3.05 ± 0.53

**Rest**				

Heart rate (beats/min)	86.5 ± 10.6	80.7 ± 8.6	87.9 ± 11.2	80.9 ± 8.8

O_2 _pulse (mL/beat)	4.43 ± 0.72	4.73 ± 0.86	4.26 ± 0.76	4.73 ± 0.88

V'O_2_/kg [mL/(min.kg)]	5.93 ± 1.28	6.27 ± 0.80	5.87 ± 1.30	6.20 ± 0.56

**Anaerobic Threshold**				

WR (Watt)	58.6 ± 24.2	82.9 ± 29.1*	54.5 ± 20.9	92.9 ± 31.1**

Heart rate (beats/min)	110 ± 14	111 ± 10	112 ± 13	118 ± 10#

O_2 _pulse (mL/beat)	7.69 ± 1.50	9.19 ± 2.42	7.01 ± 1.74 #	9.48 ± 2.69**

V'O_2_/kg [mL/(min.kg)]	12.8 ± 3.0	16.7 ± 4.0**	11.9 ± 2.9	18.0 ± 4.6**#

V'E/V'CO_2_	29.2 ± 2.3	26.9 ± 1.5**	29.1 ± 3.7	25.7 ± 2.0**#
**Maximal exercise**				

WR (Watt)	132 ± 30	188 ± 46**	125 ± 35	196 ± 51** ##

Heart rate (beats/min)	158 ± 20	167 ± 8	155 ± 21	168 ± 8*

O_2 _pulse (mL/beat)	9.19 ± 2.18	12.43 ± 5.25*	8.82 ± 2.20.	11.70 ± 3.01**

V'O_2_/kg [mL/(min.kg)]	22.3 ± 5.7	31.2 ± 7.0**	20.9 ± 5.5 ##	31.9 ± 7.4**#

^&^Data are presented as mean ± SD

Differences evaluated by multivariate or repeated measures ANOVA:

*: *P *< 0.05; ** *P *< 0.01 between patients and controls

#: *P *< 0.05; ## *P *< 0.01 between CPET1 and CPET2

The differences between the CPET2 and CPET1 are shown in table 3. The FVC, the FEV1 and the results of resting heart rate, oxygen consumption and CO_2 _production did not change in the patient and control group. At the anaerobic threshold the group of patients performed worse and the controls improved. The work rate was 4.40 ± 9.66 W less in the patient group and 7.67 ± 19.50 W higher in the control group (*P *= 0.002, 95% CI: -23.6; -0.55). Such differences were also found for the oxygen pulse (-0.67 ± 0.93 mL/beat versus 0.25 ± 1.09 mL/beat; *P *= 0.014, 95% CI: -1.68; -0.16) and oxygen uptake (-0.87 ± 1.07 mL/kg versus 1.07 ± 2.63 mL/kg; *P *= 0.001, 95% CI: -3.61; -0.26). Similar changes were found at maximal work rate: The work rate was 6.33 ± 11.5 W less in the patient group and 11.1 ± 18.3 W higher in the control group (*P *< 0.001, 95% CI: -28.8; -5.99). And the changes in the oxygen uptake were likewise (-1.33 ± 1.68 mL/kg versus 0.73 ± 1.39 mL/kg; *P *< 0.001, 95% CI: -3.22; -0.92). The improvement in the performance of the controls is likely due to the effect of training. In the group of patients the performance is the result of a similar training effect which is counteracted by the effect of the postexertional malaise.

**Table 3:** Difference between CPET2 and CPET1 in patients and controls^&^

	CPET2 minus CPET1	
	Patients n = 15	Controls n = 15

FVC (L)	0.01 ± 0.21	0.05 ± 0.18

FEV 1 (L)	0.00 ± 0.32	0.07 ± 0.17

**Rest**		

Heart rate (beats/min)	1.33 ± 6.29	0.20 ± 6.24

O_2 _pulse (mL/beat)	-0.17 ± 0.41	0.01 ± 0.47

V'O_2_/kg [mL/(min.kg)]	-0.07 ± 0.70	-0.07 ± 0.80

**Anaerobic Threshold**		

WR (Watt)	-4.40 ± 9.66	7.67 ± 19.50*

Heart rate (beats/min)	2.60 ± 7.79	4.60 ± 10.16

O_2 _pulse (ml/beat)	-0.67 ± 0.93	0.25 ± 1.09*

V'O_2_/kg [mL/(min.kg)]	-0.87 ± 1.77	1.07 ± 2.63*

V'E/V'CO_2_	-0.21 ± 2.05	-13.8 ± 50.1

**Maximal exercise**		

WR (Watt)	-6.3 ± 11.5	11.1 ± 18.3**

Heart rate (beats/min)	-3.3 ± 8.6	11.9 ± 41.6

O_2 _pulse (mL/beat)	-0.37 ± 0.70	-4.6 ± 15.0

V'O_2_/kg [mL/(min.kg)]	-1.33 ± 1.68	0.73 ± 1.39**

^&^Data are presented as mean ± SD

Differences between patients and controls evaluated by multivariate ANOVA: *: *P *< 0.05; ** *P *< 0.01

### ATP synthesis in PBMC

The results are summarized in table 4. The cells were isolated before the exercise tests. The amount of the mitochondria of PBMC was estimated by the assay of citrate synthase, and the activity was not different between the four groups (patients and controls at CEPT1 and 2).

**Table 4:** Citrate synthase activity, and complex I- and II-dependent oxidative phosphorylation in PBMC and CK in plasma of CFS patients and controls before CPET1 and CPET2^&^

	CPET1		CPET2	
	**Patients n = 15**	**Controls n = 15**	**Patients n = 15**	**Controls n = 15**

Citrate synthase (CS) U/g protein	135 ± 61	132 ± 17	128 ± 20	154 ± 51

ATP synthesis via Complex I nmol/(0.5 h. mg protein)	7.1 ± 3.1	7.8 ± 2.8	6.7 ± 4.8	9.5 ± 5.7

ATP synthesis via Complex I nmol/(0.5 h. U CS)	54 ± 19	58 ± 21	54 ± 34	61 ± 26

ATP synthesis via Complex II nmol/(0.5 h. mg protein)	7.8 ± 5.0	8.2 ± 2.8	6.8 ± 4.9	8.9 ± 5.3

ATP synthesis via Complex II nmol/(0.5 h. U CS)	58 ± 22	60 ± 26	54 ± 36	58 ± 27

CK in plasma U/L	70 ± 25	83 ± 35	64 ± 22	96 ± 63

^&^Data are presented as mean ± SD

There were no statistically significant differences between patients and controls at CPET1 or 2 (according to multivariate ANOVA), or between CEPT1 and 2 for patients or for controls (according to repeated measures ANOVA)

The ATP synthesis assayed via the reduction of complex I, expressed on basis of protein were similar in the groups, and also when the ATP synthesis rate was expressed on basis of citrate synthase. The same was found for ATP synthesis via complex II.

In the present study, plasma CK was low and not increased before and 24 h after exercise in the patient group, and not different from the control group, suggesting no muscle damage and no major intrinsic abnormalities of muscular oxidative phosphorylation in CFS/ME patients.

## Discussion

At rest the cardiopulmonary exercise test 1 and 2 showed no difference between patients and controls. Increasing work rate made the differences obvious. The lower V'O2 at the anaerobic threshold indicated that the difference in V'O2 at maximal work rate was not due to a reduced willingness to perform in the CFS/ME group.

The FEV1 and the FVC were not different, but the higher ventilatory equivalent for CO_2 _at the anaerobic threshold indicated the possibility of a ventilation-perfusion mismatch in the patient group. The reproducibility of CPET was high, relatively poor performers at the first test ranked low in the second test too. There were significant differences between the patients and controls. Based on the oxygen uptake test, the patients not only performed worse than controls in the first test, but the recovery after 24 h was not completed in this group as well. This indicates an impaired recovery [25], as expressed in the criterion "postexertional malaise" of the CDC Symptom score.

A limited mitochondrial ATP synthesis was the working hypothesis for this investigation. This is probably not true, as the energy production can be limited by other mechanisms as well. The exercise tests with increased work load suggested the possibility that the mitochondrial ATP synthesis was decreased, because the anaerobic threshold was reached. Then the mitochondria were not longer able to produce sufficient ATP to sustain the exercise, and the anaerobic glycolysis in muscle had to produce the extra ATP needed, which is reflected by the lactate production. This is also the case in patients with defects in the oxidative phosphorylation. Peripheral blood mononuclear cells are commonly used to assess the gene expression in CFS/ME [26-34], and the expressions of various genes involved in mitochondrial protein synthesis, energy metabolism, and in free radical metabolism were found to be changed. The results of the present study do not support a physiological effect of these changes, and demonstrated that the oxidative phosphorylation in PCMB of CFS/ME patients is fully normal. And it is likely that also their muscle mitochondria are normal, since 24 h after strenuous exercise CK did not leak to the blood, as is the case in patients with defective oxidative phosphorylation.

A recent publication [6] claimed to have found a defective oxidative phosphorylation in neutrophils of CFS/ME patients, but the flux through this process had not been measured. These investigators performed a so called "ATP profile" test, and determined ATP under five different conditions, and the sum of these was found to be abnormal in 70 of 71 patients. One of us (WS) was involved in an investigation that clearly showed that neutrophils do not catalyze oxidative phosphorylation and the remaining complexes of the respiratory chain maintain the mitochondrial membrane potential [35]. Their mitochondria are only active in apoptosis [36].

In line with the present work, Mathew et al [37] discovered by proton magnetic resonance spectroscopy imaging that ventricular cerebrospinal lactate was 3.5-fold increased in CFS/ME patients. McCully and Natelson [38] demonstrated by combined near-infrared spectroscopy and ^31^P magnetic resonance spectroscopy that during exercise the oxygen delivery to skeletal muscle was delayed. Neary et al [39] established by near-infrared spectrometry that the oxygenation of the prefrontal brain lobe was decreased in exercising patient to 67% of that in controls, confirming the results of a study by Streeten and Bell [40] and Hurwitz et al [41] and in line with a decreased blood volume in CFS patients.

## Conclusions

The decrease in mitochondrial ATP production at increasing work rate, detected by the CPET tests in the present well-characterized though small group of CFS/ME patients, is a secondary phenomenon. This was shown by the normality of the oxidative phosphorylation in peripheral blood mononuclear cells. The chain of mechanisms that couple (external) pulmonary to (internal) cellular respiration showed no abnormal differences in this study between CFS patients and healthy controls at the pulmonary, cardiac and circulatory level. Two possible explanations for the insufficient energy production in CFS remained: a lower transport capacity of oxygen as in anemia or a mitochondrial insufficiency. We showed that the mitochondrial ATP production shows no defect. Then the conclusion must be that the transport capacity of oxygen is limited in CFS patients.

## Abbreviations

CDC: US Centers for Disease Control and Prevention; CFS/ME: chronic fatigue syndrome/myalgic encephalopathy; CK: creatine kinase; CPET: cardiopulmonary exercise test; CS: citrate synthase; FEV_1: _forced expiratory volume in the first second; FVC: forced vital capacity; PBMC: peripheral blood mononuclear cells; V'CO_2_: carbondioxyde output; V'E: minute ventilation; V'O_2_: oxygen uptake; WR work rate

## Competing interests

The authors declare that they have no competing interests.

## Authors' contributions

All authors were involved in the set up of the experiments. The clinicians (RCWV, RMK, FCV) performed the clinical experiments. The blood separation and the biochemical assays were done by WS. The first drafts of the paper were written by RCWV and HRS. The final manuscript was read and approved by all authors.
